# Interpersonal violence against people with intellectual disabilities in São Paulo, Brazil: characteristics of victims, perpetrators and referrals

**DOI:** 10.1186/s12889-024-19211-4

**Published:** 2024-07-05

**Authors:** Mariana Teixeira da Silva, Ana Vitória Bastos Fontoura, Agatha Nicoly Guedes Pires, Ana Paula Pinho Carvalheira, Paula Hino, Meiry Fernanda Pinto Okuno, Mônica Taminato, José Manuel Peixoto Caldas, Hugo Fernandes

**Affiliations:** 1 Department of Public Health, Federal University of São Paulo, São Paulo, Brazil; 2 Department of Nursing, São Paulo State University, Botucatu, Brazil; 3 Faculty of Law, University of Salamanca, Salamanca, Spain

**Keywords:** Intellectual disability, Epidemiology, Violence, Health surveillance, Public health

## Abstract

**Introduction:**

Interpersonal violence is a phenomenon that can occur with different people and conditions. However, people with intellectual disabilities have increased vulnerability to this problem, with potential risks to their health and well-being. The aim of this study was to identify the sociodemographic characteristics of people with disabilities who have been victims of interpersonal violence, the profile of the perpetrators and the measures taken after the victims have been cared for.

**Methods:**

This is an exploratory, descriptive, cross-sectional study using the Interpersonal Violence Notification Forms entered into the Brazilian Ministry of Health’s Notifiable Diseases Information System. The city of São Paulo was chosen as the setting because it is the largest city in Latin America and has a faster data processing system than other cities. The period covered notifications made between 2016 and 2022. The information was collected between October and November 2023 and a univariate statistical analysis was carried out. Fisher’s exact test was used, with a significance level of 5% (*α* = 0.05).

**Results:**

There were 4,603 notifications against people with intellectual disabilities in the period. The forms of physical violence, neglect/abandonment and psychological/moral violence were more frequent in the 15–19 age group, while sexual violence was more frequent in the 10–14 age group (*p* < 0.001). The sex most often attacked was female in all the forms investigated (*p* < 0.001) and the skin colors of the most victimized people were black and/or brown, except in cases of neglect/abandonment (*p* = 0.058). Most of the victims had little schooling (*p* = 0.012). The aggressions were committed by one person (*p* < 0.001), known or related to the victim, such as mother or father, except in cases of sexual violence, where strangers were the main perpetrators (*p* < 0.001). The sex of the perpetrator was male, except in cases of neglect and/or abandonment (*p* < 0.001), and the age was between 25 and 29 (*p* = 0.004). In cases of sexual violence, rape was the most frequent and the procedures carried out were blood collection followed by prophylaxis for Sexually Transmitted Infections (STIs) were the main procedures carried out by health professionals (*p* = 0.004). The majority of referrals made after receiving care were to the health and social assistance network, with few referrals to bodies such as the human rights reference center, guardianship council and police stations (*p* < 0.001).

**Conclusion:**

People with intellectual disabilities are highly vulnerable to the forms of violence studied, especially children and adolescents, black or brown, with low levels of education. The perpetrators are usually close people, male and older than the victims. The referrals made by health professionals did not prioritize the victim’s safety and the guarantee of human rights. Lines of care for the health of victims of violence should be implemented, taking into account special aspects, such as people with intellectual disabilities, whose search for help can be difficult.

## Introduction

Intellectual disability can be understood as below-average intellectual functioning, appearing before the age of 18, accompanied by limitations in adaptive functioning in at least two of the following areas: communication, self-care, domestic life, social/interpersonal relationships, use of community resources, self-sufficiency, academic skills, work, leisure, health and safety [1]. It is estimated that there are more than 2,000 clinical conditions in which intellectual disability is one of the most important manifestations. The challenge of social inclusion for the intellectually disabled depends on guaranteeing their rights to health and the prevention of health problems, and the impact of mental health problems can significantly reduce their possibilities in this area [1, 2].

Limitations in adaptive functioning associated with prejudice, family difficulties, scarce access to specialized services and the slow implementation of appropriate public policies can be important components of the vulnerability of people with intellectual disabilities to conditions of violence. Violence against people with intellectual disabilities is a serious and historically recurring phenomenon in humanity. In different cultures and regions of the world, people with intellectual disabilities have been victims of different forms of aggression such as sexual, psychological and/or moral and physical [2, 3].

A Brazilian study on compulsory notifications of interpersonal violence found that violence against vulnerable populations, such as the physically and intellectually disabled, is recurrent and potentially threatens their integrity and dignity [4]. The indication of an intellectual disability is also cited as an important indicator in compulsory notifications of violence, as it helps to direct more efficient public policies to combat this problem, since it may be motivated by prejudice or strong manifestations of power [4, 5].

However, the phenomenon is not restricted to developing countries. A scoping review on the mistreatment of people with disabilities carried out in the United States found that in the studies published between 1995 and 2005 on the subject, there was a higher prevalence of mistreatment of people with intellectual disabilities when compared to people with other types of disability. Furthermore, the abuse tended to be much more serious in this population than in others, causing significant damage to the quality of life of victims and witnesses [5].

In searches of the main health databases, few robust epidemiological studies were identified that characterized the victims, perpetrators and outcomes of aggression, especially in Latin American countries. Thus, the relevance of this study lies in providing an analysis of interpersonal violence against people with intellectual disabilities, in order to support good intersectoral practices and/or public policies for the prevention and control of violence. The aim of this study was to identify the sociodemographic characteristics of people with intellectual disabilities who are victims of interpersonal violence, the profile of the perpetrators and the measures taken after the victims have been treated.

## Methods

This is an exploratory, descriptive, cross-sectional study. The research was guided by the *Strengthening the Reporting of Observational Studies in Epidemiology* (STROBE) tool [6] .

### Study protocol

The categories chosen were: physical, psychological and/or moral violence, sexual violence, neglect and/or abandonment and torture. The following variables were investigated in relation to the victims in each of the categories: age group, gender, skin color, schooling, marital status, sexual orientation and gender identity. In relation to the perpetrator, the following variables were investigated: number of people involved, relationship and/or degree of kinship, gender of the probable perpetrator of the violence, life cycle and suspected use of alcohol. Finally, the variables collected about the occurrence were: location, whether it had happened on other occasions, motivation, means of aggression and referrals made after the victims had been attended to. In cases of sexual violence, the variables type of violence and procedure carried out were added.

Data was collected using the National Notification Form for Suspected or Confirmed Cases of Interpersonal Violence from the Notifiable Diseases Information System (SINAN). The forms were entered into the information system by the notifying health units, such as Epidemiological Surveillance, Ambulatory Medical Care (AMA) units and other public primary health care, outpatient (general and specialized) and hospital services with SINAN accreditation. Subsequently, the data was made available by the National Health Surveillance System (SNVS) through the TabNet program, developed by the Department of Information Technology of the Unified Health System (DATASUS) and available on the internet without restriction.

### Data collection

The data was extracted from the TabNet system of the São Paulo Municipal Health Department (SMS). São Paulo was chosen as the research setting because it is the largest city in Brazil and Latin America, with more than 11 million inhabitants from all regions of the country. In addition, national data had only been tabulated up to 2019 at the time of collection and may not reflect the real epidemiological situation [7]. To collect the data, we used the internet to access the program on the SMS website, available at: http://tabnet.saude.prefeitura.sp.gov.br/cgi/deftohtm3.exe?secretarias/saude/TABNET/SINAN/RVI*OLE/RViolenciaNet.def*.

The period outlined for this study was the notification of cases of violence between 2016 and 2022. The period prior to 2016 was not included due to changes in the compulsory notification form in 2015. The data for 2023 had not yet been tabulated by the system, so all occurrences were not accessible. The information was collected between October and November 2023, and the data was sorted using the Excel 365 program. The following inclusion criteria were adopted: notifications of suspected or confirmed cases of interpersonal violence of the physical, psychological and/or moral, sexual, neglect and/or abandonment and torture types against people with intellectual disabilities treated in health services in São Paulo, capital. Notifications of cases of violence with ‘ignored’ records in the field of intellectual disability and violence of the types of human trafficking, financial/economic, child labor, legal intervention and “other” were excluded. These exclusions were justified by the fact that the records did not meet the objective of the study and the inclusion criteria.

### Ethical aspects

As these were studies carried out on existing databases, it was not necessary to seek approval from the Research Ethics Committee. All stages of the study complied with national and international research ethics standards. In compliance with resolution 200/2021 of the University Council of the Federal University of São Paulo (Unifesp), a Declaration of Responsibility was signed by the head of the Department of Collective Health at Unifesp to guarantee the confidentiality of the results obtained from public secondary data. Finally, the data obtained was stored on a hard disk, with access protected by a double-check password, under the possession of the main researcher.

### Analysis

Univariate statistical analysis was carried out using R software, version 4.2.2. As the data set consisted of categorical variables, a descriptive analysis of the data was carried out using simple absolute and percentage frequencies. Fisher’s exact test was used to verify associations between type of violence and other variables (motivation for violence, means of aggression, whether sexual violence occurred, place of occurrence, sex of perpetrator, life cycle of aggressor, referral, age group, race, schooling and marital status) when the expected value of the matrix was less than five. The significance level was estimated at 5% (*α* = 0.05). As the data was tabulated by the Unified Health System information system, with no unit information, it was not possible to make adjustments that would be necessary for logistic regression, data imputation or other tests. All the terms and intervals (such as ages) were the same as those presented by the TabNet system and present in the National Notification Form for Suspected or Confirmed Cases of Violence. The data collected in this study can be consulted at https://osf.io/4h68x/.

## Results

During the period in question, there were 4,603 notifications against people with intellectual disabilities in São Paulo, SP. There was a statistical association between the variables age group, skin color, gender, schooling, marital status and sexual orientation, as shown in Table 1.

**Table 1:** Notifications of violence against people with intellectual disabilities according to the victim’s sociodemographic characteristics, São Paulo, Brazil, 2016–2022

Type of violence						
Variable	Physics	Neglect/Abandonment	Torture	Psychological/ Moral	Sexual	*p*-value*
	*n* (%)	*n* (%)	*n* (%)	*n* (%)	*n* (%)	
**Age group (years**)						
0 a 4	44(2,4)	74(8,4)	3(3,4)	27(2,8)	34(4,2)	<0,001
5 a 9	78(4,2)	87(9,8)	6(6,8)	70(7,2)	83(10,2)	
10 a 14	184(10)	93(10,5)	8(9,1)	137(14,1)	179(22)	
15 a 19	255(14)	117(13,3)	6(6,8)	171(17,6)	153(18,8)	
20 a 24	198(10,7)	66(7,5)	12(14)	79(8,1)	105(13)	
25 a 29	184(10)	33(3,7)	5(5,7)	71(7,3)	75(9,2)	
30 a 34	188(10,2)	30(3,4)	13(14,7)	71(7,3)	56(6,9)	
35 a 39	152(8,2)	39(4,4)	7(7,9)	72(7,4)	32(3,9)	
40 a 44	138(7,5)	26(3,0)	6(6,8)	59(6,1)	25(3,1)	
45 a 49	124(6,7)	37(4,2)	6(6,8)	61(6,3)	19(2,3)	
50 a 54	82(4,4)	22(2,5)	4(4,5)	39(4)	17(2,1)	
55 a 59	76(4,1)	29(3,3)	3(3,4)	29(3,0)	13(1,6)	
60 a 64	55(3,0)	46(5,2)	4(4,5)	31(3,2)	8(1)	
65 a 69	30(1,6)	36(4,1)	1(1,1)	11(1,1)	7(0,8)	
70 a 74	14(0,7)	37(4,2)	0(0)	13(1,3)	4(0,5)	
75 and more	48(2,6)	109(12,4)	4(4,5)	31(3,2)	3(0,4)	
**Sex**						
Male	767(41,5)	410 (46,5)	36 (41)	326(33,6)	172(21,2)	< 0,001
Female	1083(58,5)	471(53,5)	52 (59)	645(66,4)	641(78,8)	
**Skin color**						
Ign /White^#^	99(5,4)	45(5,1)	7(8)	56(5,7)	44(5,4)	0,058
White	785(42,4)	416(47,2)	34(38,6)	399(41)	342(42,1)	
Black / Brown	951(51,4)	399(45,4)	47 (53,4)	503(51,8)	414(50,9)	
Yellow	12(0,6)	18(2,0)	0(0)	10(1,0)	8(1,0)	
Indigenous	3(0,2)	3(0,3)	0	4(0,5)	5(0,6)	
**Education**						
Ign /White^#^	575(31,1)	301(34,2)	29(33)	264(27,2)	231(28,4)	0,012
Illiterate	112(6,0)	106(12)	13(14,7)	69(7,1)	51(6,3)	
1stto4thgrade incomplete^**^	220(11,9)	128(14,5)	10(11,4)	123(12,6)	85(10,5)	
Complete 4th grade^**^	84(4,5)	28(3,2)	2(2,3)	45(4,6)	31(3,8)	
5thto8thgrade incomplete^**^	262(14,2)	86(9,7)	12(13,6)	153(15,7)	146(17,9)	
Completeprimary education	107(5,8)	35(3,9)	4(4,5)	56(5,7)	33(4,0)	
High school incomplete	188(10,2)	51(5,8)	4(4,5)	105(10,8)	92(11,3)	
Completed high school	183(9,9)	40(4,5)	6(6,8)	85(8,7)	64(7,8)	
Incompletehigher education	35(1,9)	1(0,1)	1(1,1)	15(1,5)	11(1,4)	
Completehigher education	20(1,1)	3(0,3)	3(3,4)	10(1,0)	9(1,1)	
Not applicable	64(3,5)	102(11,6)	4(4,5)	47(4,8)	60(7,4)	
**Marital status**						
Blank/Ignored/Not applicable						<0,001
	421(22,7)	324(36,8)	17(19,3)	230(23,6)	237(29,5)	
Single	1024(55,4)	400(45,4)	48(54,5)	524(53,9)	509(62,6)	
Married/Consensual Union	270(14,6)	62(7,0)	16(18,2)	133(13,7)	35(4,3)	
Widowed	51(2,7)	73(8,3)	4(4,5)	47(4,8)	4(0,5)	
Separate	84(4,5)	22(2,5)	3(3,4)	38(3,9)	18(2,2)	
**Sexual orientation**						
Heterosexual	987(53,3)	299(34)	46(52,3)	495(51)	348(42,8)	< 0,001
Homosexual	33(1,8)	10(1,1)	1(1,1)	12(1,2)	15(1,8)	
Bisexual	25(1,4)	4(0,4)	1(1,1)	15(1,5)	12(1,5)	
Not applicable	288(15,6)	279(31,7)	16(18,2)	205(21,1)	252(31)	
Ignored	517(27,9)	289(32,8)	24(27,3)	245(25,2)	186(22,9)	
**Gender identity**						
Transvestite	6(0,3)	4(0,5)	0	2(0,2)	2(0,2)	0,866
Transsexual woman	15(0,8)	6(0,7)	1(1,1)	11(1,1)	6(0,7)	
Transsexual man	5(0,3)	2(0,2)	0	1(0,1)	2(0,2)	
Not applicable	1249(67,5)	606(68,8)	55(62,5)	673(69,2)	605(74,4)	
Ignored	575(31,1)	263(29,8)	32(36,4)	285(29,3)	198(24,4)	

*Source* Notifiable Diseases Information System

*Fisher’s exact test. ^#^Ignored. ^**^Primary education

Physical, neglect/abandonment and psychological/moral violence were more frequent in the 15–19 age group (14%, *n* = 255; 13.3%, *n* = 117 and 17.6%, *n* = 171) (*p* < 0.001). Torture was more common among people aged between 30 and 34 (14.7%, *n* = 13) and sexual violence was more common among people aged between 10 and 14 (22%, *n* = 179). The sex most affected was female in all the forms investigated (*p* < 0.001) and the skin color of the most victimized people was black and/or brown, except in cases of neglect/abandonment (47.2%, *n* = 416) (*p* = 0.058).

As for schooling, there was a divergence according to the manifestation of violence. People with between 5th and 8th grade education were the main victims of physical violence (14.2%, *n* = 262), psychological/moral violence (15.7%, *n* = 153) and sexual violence (17.9%, *n* = 146). On the other hand, cases of neglect/abandonment were more frequent among those with incomplete primary schooling (14.5%, *n* = 128) and cases of torture among illiterate people (14.7%, *n* = 13). However, for all types of violence, there was a high number of notifications with an unknown/white response for the victims’ schooling (*p* = 0.012).

The prevalent marital status was single in all types of violence (*p* < 0.001), as well as heterosexual orientation (*p* < 0.001). There was no significant association with gender identity.

The characteristics of the perpetrator according to the type of violence are shown in Table 2.

**Table 2:** Notifications of violence against people with intellectual disabilities according to the characteristics of the perpetrator, São Paulo, SP, Brazil, 2016-2022

Type of violence								
Variable		Physics	Neglect/Abandonment	Torture	Psychological/ Moral	Sexual	*p*-value*	
		*n* (%)	*n* (%)	*n* (%)	*n* (%)	*n* (%)		
**Number of people involved**								
One		1282(70)	408(47,1)	54(62,8)	600(62,4)	600(74)	< 0,001	
Two or more		443(24,1)	366(42,3)	27(31,4)	296(30,7)	145(18)		
Ignored		108(5,9)	92(10,6)	5(5,8)	66(6,9)²	65(8)		
**Relationship**								
Dad		173(8,5)	181(15,4)	7(7,2)	157(13)	81(9,7)	<0,001	
Mother		205(10)	345(29,3)	17(17,5)	178(14,7)	15(1,7)		
Stepfather		44(2,1)	26(2,2)	2(2,1)	36(3)	57(6,8)		
Stepmother		17(0,8)	13(1,1)	1(1,0)	8(0,6)	1(0,1)		
Spouse		182(9)	34(2,9)	6(6,2)	95(7,8)	23(3)		
Ex-spouse		40(2)	11(0,9)	5(5,1)	24(2)	6(0,7)		
Boyfriend		43(2,1)	2(0,2)	2(2,1)	16(1,3)	11(1,3)		
Ex-boyfriend		11(0,5)	2(0,2)	0(0)	10(0,8)	7(0,8)		
Child		101(5)	161(13,7)	5(5,1)	75(6,2)	3(0,3)		
Brother		180(9)	110(9,4)	10(10,4)	96(8)	40(4,7)		
Friend/acquaintance		270(13,2)	41(3,5)	11(11,3)	151(12,5)	245(29,3)		
Unknown		227(11,1)	19(1,6)	11(11,3)	90(7,4)	199(23,7)		
Caregiver		37(1,8)	67(5,7)	2(2,1)	30(2,5)	9(1,0)		
Boss		3(0,1)	1(0,1)	0	2(0,2)	4(0,5)		
Person with institutional relationship		42(2)	17(1,4)	1(1)	32(2,6)	23(3)		
Police/law enforcement		17(0,8)	1(0,1)	1(1)	1(0,1)	1(0,1)		
Own person		313(15,4)	65(5,5)	6(6,2)	109(9)	8(0,9)		
Other links		134(6,6)	80(6,8)	10(10,4)	100(8,3)	104(12,4)		
**Sex of the probable perpetrator**								
Male		1102(59,6)	212(24,2)	60(68,2)	545(56,2)	712(87,6)	< 0,001	
Female		496(26,8)	309(35,2)	20(22,7)	219(22,6)	24(2,9)		
Both sexes		111(6)	232(26,5)	4(4,5)	127(13,1)	14(1,7)		
Ignored		139(7,5)	124(14,1)	4(4,5)	79(8,1)	63(7,7)		
**Life cycle of the probable perpetrator of violence**								
Children (0–9 years)	32(1,7)		11(1,3)	1(1,1)	10(1,0)	13(1,6)		0,004
Teenagers (10–19 years)	218(11,8)		44(5)	6(6,8)	111(11,4)	117(14,4)		
Young (20–24 years)	204(11)		56(6,4)	12(13,6)	71(7,3)	65(8)		
Adult (25–59 years)	1002(54,2)		500(56,7)	47(53,4)	568(58,5)	405(49,8)		
Elderly person (60 years or older)	105(5,7)		75(8,5)	8(9,1)	70(7,2)	52(6,4)		
Ignored	289(15,6)		195(22,1)	14(16)	142(14,6)	161(19,8)		
**Suspected alcohol use**								
Yes	473(25,6)		120(13,7)	29(32,9)	224(23,1)	172(21,2)		< 0,001
No	818(44,3)		453(51,9)	40(45,5)	445(46)	305(37,5)		
Ignored	557(30,1)		300(34,4)	19(21,6)	300(30,9)	336(41,3)		

Source: Notifiable Diseases Information System

*Fisher’s exact test

In all types of violence, the number of perpetrators involved was one person (*p* < 0.001). The relationship or degree of kinship with the victim varied. In cases of physical violence, the person themselves caused the aggression (15.4%, *n* = 313), followed by a friend or acquaintance (13.2%, *n* = 270). Neglect and/or abandonment was caused by the mother (29.3%, *n* = 345) and father (15.4%, *n* = 181), as was psychological and/or moral violence (14.7%, *n* = 178; 13%, *n* = 157). Cases of torture were perpetrated by friends or acquaintances (11.3%, *n* = 11) and an equal number by strangers. The latter were also the main perpetrators of sexual violence (29.3%, *n* = 245; 23.7%, *n* = 199) (*p* < 0.001). It was not possible to analyze whether there was any conflicting relationship between victim and perpetrator as the instrument did not include this question.

The sex of the perpetrator was male in most types of violence, with the exception of neglect and/or abandonment, where the perpetrator was female (35.2%, *n* = 309) (*p* < 0.001). The perpetrator’s life cycle was the same for all types of violence, with adults aged between 25 and 59 (*p* = 0.004). Suspected alcohol consumption was predominantly negative in the forms of violence investigated (*p* < 0.001).

The characteristics of the violence and the referrals made by the professionals after receiving care are described in Table 3.

**Table 3:** Notifications of violence against people with intellectual disabilities according to characteristics of the violence and referrals made, São Paulo, SP, Brazil, 2016-2022

Type of violence						
Variable	Physics	Neglect/Abandonment	Torture	Psychological/ Moral	Sexual	*p*-value*
	n (%)	n (%)	n (%)	n (%)	n (%)	
Residence	1253(67,7)	716(80,4)	53(60,2)	716(73,7)	453(55,8)	< 0,001
Collective housing	35(1,9)	11(1,2)	3(3,4)	13(1,3)	19(2,3)	
School	49(2,6)	11(1,2)	1(1,1)	37(3,8)	40(4,9)	
Sports venue	6(0,3)	0	1(1,1)	1(1,0)	2(0,2)	
Bar or similar	17(0,9)	2(0,2)	2(2,3)	7(0,7)	9(1,1)	
Public roads	251(13,6)	27(3,0)	11(12,5)	64(6,6)	94(11,6)	
Trade/Services	32(1,7)	14(1,6)	3(3,4)	17(1,7)	19(2,3)	
Industries/construction	1(0,05)	1(0,1)	0	3(0,3)	2(0,2)	
Others	101(5,5)	44(4,9)	4(4,5)	59(6,1)	78(9,6)	
Ignored	105(5,7)	65(7,3)	10(11,4)	54(5,6)	95(11,7)	
**It happened other times**						
Blank	8(0,4)	6(0,7)	0	2(0,2)	5(0,6)	< 0,001
Yes	1105(59,7)	572(70,5)	60(68,2)	680(70)	380(46,7)	
No	450(24,3)	100(12,3)	20(22,7)	168(17,3)	274(33,7)	
Ignored	287(15,5)	203(25)	8(9,1)	122(12,5)	154(19)	
**Motivation**						
Sexism	149(8)	11(1,2)	7(7,9)	115(11,8)	242(30)	< 0,001
Homophobia/Lesbophobia Bi phobia/Transphobia	9(0,5)	7(0,5)	3(3,4)	4(0,4)	1(0,1)	1(0,1)
Racism	9(0,5)	0	0	1(0,1)	0	
Religious intolerance	0	1(0,1)	0	0	0	
Xenophobia	2(0,1)	0	0	1(0,1)	0	
Generational conflict	241(13)	74(8,4)	6(6,8)	132(13,6)	10(1,2)	
Street situation	50(2,7)	12(1,4)	2(2,3)	21(2,2)	18(2,2)	
Disability	166(9)	123(13,9)	10(11,4)	98(10,1)	72(8,8)	
Others	517(27,8)	237(27)	24(27,3)	225(23,1)	119(14,6)	
Not applicable	193(10,4)	154(17,5)	12(13,6)	142(14,6)	141(17,3)	
Ignored	520(28)	262(30)	24(27,3)	233(24)	210(25,8)	
**Means of aggression**						
Body strength/thrust	1286(55,5)	105(19,5)	57(40,7)	442(41,2)	370(53,2)	< 0,001
Hanging	93(4)	7(1,3)	11(8)	43(4)	11(1,5)	
Blunt object	142(6,1)	17(3,1)	13(9,3)	39(3,6)	5(0,7)	
Sharps	202(9)	22(4,1)	14(10)	71(6,6)	20(3)	
Hot substance/object	34(1,5)	6(1,1)	1(0,7)	6(0,6)	0	
Poisoning/Intoxication	92(3,9)	15(2,8)	0(0)	24(2,2)	9(1,3)	
Firearms	12(0,5)	1(0,2)	3(2,1)	14(1,3)	7(1,0)	
Threats	316(13,6)	87(16,2)	31(22,1)	321(30)	180(25,8)	
Other means	138(5,9)	279(51,7)	10(7,1)	112(10,5)	94(13,5)	
**If sexual violence, what kind?**						
Sexual harassment					259(26,6)	0,004
Rape					603(61,9)	
Pornography					18(1,8)	
Sexual exploitation					40(4,1)	
Other sexual violence					54(5,5)	
**Procedure carried out**						
STI prophylaxis^#^					185(19)	0,004
HIV prophylaxis**					179(18,4)	
Hepatitis B prophylaxis					148(15,2)	
Blood collection					186(19,1)	
Semen collection					26(2,7)	
Vaginal secretion collection					41(4,2)	
Emergency contraception					81(8,3)	
Legal abortion					17(1,7)	
Others					111(11,4)	
**Referral**						
Health Network	1284(49)	696(41,6)	62(38,8)	704(41,4)	518(36,1)	< 0,001
Social assistance network	351(13,6)	427(25,5)	27(16,8)	297(17,5)	201(14)	
Education Network	40(1,5)	37(2,2)	3(2)	45(2,6)	13(1)	
Women’s Service Network	167(6,5)	20(1,2)	15(9,4)	140(8,2)	204(14,2)	
Guardianship Council	260(10)	258(15,4)	12(7,5)	215(12,6)	219(15,3)	
Council for the elderly	33(1,3)	72(4,3)	6(3,7)	30(2)	6(0,4)	
Police Station for the Elderly	20(1)	26(1,6)	4(2,5)	20(1,2)	2(0,1)	
Human Rights Reference Center	14(0,5)	15(0,9)	1(0,6)	6(0,3)	4(0,3)	
Public Prosecutor’s Office	29(1,1)	46(3)	2(1,2)	24(1,4)	8(0,6)	

Source: Notifiable Diseases Information System

*Fisher’s exact test. ^#^Sexually Transmitted Infections.^**^ Human Immunodeficiency Virus

The location of all types of violence against people with intellectual disabilities was their home (*p* < 0.001) and it happened more than once (*p* < 0.001). Motivation varied, with cases of physical violence (28%, *n* = 520), neglect and/or abandonment (30%, *n* = 262) and psychological and/or moral violence (24%, *n* = 233) being reported as ignored. Cases of sexual violence were motivated by sexism (30%, *n* = 242) and torture by others (27.3%, *n* = 24) (*p* < 0.001).

In cases of sexual violence, the most frequent manifestation was rape (61.9%, *n* = 603) and the procedures carried out were blood collection (19.1%, *n* = 186) followed by prophylaxis for Sexually Transmitted Infections (STI) (19%, *n* = 185) (*p* = 0.004).

The referrals made by the professionals after assisting the victims were in the majority of cases (in all typifications) to the health and social assistance network, with low referrals to bodies such as the human rights reference center, guardianship council and police stations (*p* < 0.001).

## Discussion

Violence against people with intellectual disabilities is a broad, highly complex issue that generates debate when it is addressed [8]. This is understandable when we consider that violence encompasses various factors that increase the vulnerability of victims, among them age. Age as a social marker suggests a global tendency for adolescents and young people to be vulnerable to interpersonal violence, which is one of the main causes of premature death, injury and disability among this demographic group [9]. It is estimated that more than 200,000 deaths occur each year among the 10–29 age group, with homicides ranking fourth among the causes of death [9, 10]. Added to this is the susceptibility to acts of violence against young people and children, especially people with disabilities, who are around 70% more likely to be abused than other people [10].

Therefore, children and young people with disabilities face higher rates of abuse, including sexual abuse, compared to their peers without disabilities. A meta-analysis containing 16,831,324 children cites that those with disabilities had a high overall prevalence of violence (31.7%), with a probability of occurrence 2.08 times higher than in children without any type of disability^(10)^. This is corroborated by the data in the table, which shows a high incidence of sexual violence among people between the ages of 10 and 19, but it should be borne in mind that there were a significant number of cases described as “ignored” or “blank” in relation to age and schooling, which makes it difficult to accurately state the age group most affected, as well as the schooling of the victims, which can lead to a distortion of reality.

Researchers say that incompleteness (filling in fields such as “ignored” or left “blank”) when filling in compulsory notification forms causes damage to the proper planning of actions and measures to tackle diseases and illnesses, requiring constant training for notifiers and that these failures occur frequently in various types of illnesses and diseases, being justified by haste in filling in the document or by the notifier not considering that data as relevant at the time of care or consultation [11]. However, it is clear that people with low levels of education are more prone to interpersonal violence and often have less knowledge about the health services and protection they are entitled to.

With regard to the sex of the victim, it can be seen that for all types of violence, the proportion of female notifications was higher than the proportion of male notifications. In view of this, it is worth pointing out that gender inequality is one of the main social problems today. The creation and strengthening of patriarchy as a dominant social structure plays a fundamental role in this dynamic, since it privileges men over women, based on norms and practices associated with gender performance. These norms have been internalized and reproduced throughout history, influencing the perception of masculinity and femininity, in order to justify the acts of violence that become necessary to maintain or recover the socially established gender boundaries, providing the maintenance of male privileges [12]. It is also worth noting that in this study, heterosexual people appear to have been more victims of violence than those of other orientations. However, it is necessary to reflect that machismo is a structural phenomenon throughout the world, especially in Latin American countries, which can lead many people to self-declare as heterosexual in order to avoid exposure and judgment, even by health professionals [13].

With regard to marital status, although the prevalent situation in the data presented is single, the high number of victims in a consensual/married union is also relevant. Women with intellectual disabilities are more vulnerable to partner violence and other forms of abuse than women without comorbidities. Specifically, in the context of sexual violence, these women face various barriers related to sex education, including lack of knowledge, limited access to health care and social isolation and, as a result, are at greater risk of sexual violence [12, 13].

More violence against people with brown or black skin signals an important feature of structural racism in society, marked by social inequalities, serious insults and denial of the pain and suffering of black people^(14)^. Its effects are felt in various conditions of intersectionality between race/color and health, such as in cases of violence. The findings indicate that black and/or brown people are more vulnerable to violence, as in other studies that have sought to establish epidemiological profiles of this problem in Brazil [14, 15]. It should be noted that the Brazilian Institute.

of Geography and Statistics (IBGE) considers brown people to be those with phenotypic traits of miscegenation between white and black people, black and indigenous people and indigenous people with white people, usually having a less dark skin color. Black people, on the other hand, are those with darker skin, with little or no phenotypic traits of miscegenation between people with different skin colors. Despite Brazil’s great miscegenation, there is a higher concentration of brown and black people in the north and northeast of the country [15].

The type of relationship between the perpetrator and the victim varies from case to case, and can include family members, friends, acquaintances or even strangers. One risk characteristic, which is not necessarily linked to the dynamic between the victim and the aggressor, is the constant need for victims to receive help from the perpetrators with activities of daily living, such as getting out of bed and eating, for example [16, 17].

A study carried out in Spain with 260 people with intellectual disabilities found that 59.2% of the sample had suffered physical or verbal abuse or neglect from their caregiver [16], although the link or degree of kinship referred to as “Caregiver” in the table of characteristics of perpetrators is not the most prevalent in the characteristics of perpetrators, the role of caregiver can be taken on by a family member or another person with direct social ties, such as friends or acquaintances. Thus, the main caregivers of people with intellectual disabilities are, for the most part, these same individuals with the closest social ties. Consequently, when an aggressor is also the caregiver, the harmful effect of the abuse is intensified by the victim’s dependence [17, 18]. An interesting piece of information in this study would be to investigate whether there were conflicting relationships between those involved (victim and aggressor), but the notification form did not contain this type of information.

With regard to the significant proportion of physical aggression caused by the person themselves, authors cite a possible association between intellectual disabilities and self-inflicted aggressive behavior, since self-injurious behavior is more prevalent in this population than in the general population^(19–20)^. This behavior, regardless of whether it is observed in people with or without intellectual disabilities, is a cause for concern. It indicates suffering and a need for support, as well as being a signpost for the development of additional mental health problems, such as the risk of suicide. The cause of self-violent behavior in people with intellectual disabilities can vary, such as attempts to communicate something, or the display of anger or pain, both of which are not adequately addressed by family members or guardians. Depending on how the social circle reacts, this self-harm behavior is stimulated, leading the person to repeat it periodically [19].

Still within this sphere, as young people, people with intellectual disabilities may experience more neglect or abandonment by their social circle [20], converging to the same data presented in the.

table, which is the high rates of the type of violence “neglect and/or abandonment” being mostly provoked by the mother followed by the father. The birth of a child with a disability can trigger divorce and/or parental neglect on the part of one of the parents [21]. However, evidence indicates that children with some degree of intellectual disability tend to be abandoned by the father, while mothers report that they are the ones who are abandoned or subject to divorce by the parent [21, 22].

Furthermore, considering the maternal figure as the main caregiver, the existence of dysfunctions in family dynamics, inadequate coping strategies, and also women’s work overload not only adversely influence family dynamics, but also contribute to the deterioration of the mother’s physical, mental and social health [22, 23]. This may justify mothers’ violent impulses against their children with intellectual disabilities, especially in times of great tension or stress.

As found in a study carried out in Norway [8], in cases of sexual violence, people with intellectual disabilities are often unable to quickly identify signs of harassment or abuse, and the aggressors know this and take advantage of moments when the victim is alone, usually at home, or without possible witnesses nearby. Threats and/or forms of “bargaining” by the perpetrator prevent victims from reporting the abuse, contributing to further occurrences of the offense^(8,10)^, as identified in this study.

The perceived ease with which people with intellectual disabilities are attacked means that perpetrators quickly move on from situations of harassment to rape. Researchers from the United Kingdom [24] highlight the importance of family members, health professionals and other professionals involved in the daily lives of people with intellectual disabilities keeping a constant eye out for changes in behavior, irritability, fear, body marks and other signs and symptoms that could indicate sexual violence. In addition, post-exposure prophylaxis protocols should be implemented quickly in cases of suspected sexual violence, such as rapid testing, updating vaccinations such as Hepatitis B, and offering medication to prevent Sexually Transmitted Infections and HIV infection [25, 26].

A scoping review carried out by Australian researchers, which aimed to identify the main strategies for preventing violence against people with intellectual disabilities, suggests that specific lines of care for people with intellectual disabilities who are victims of violence can contribute positively to reducing the problem and to secondary prevention [27]. In other words, the use of general lines of care may not help health professionals to take assertive measures for this population, especially those that guarantee human rights, a fact that was evidenced in this study, where there was a low number of referrals to social security, human rights and guardianship councils (in the case of children).

### Limitations and implications for the advancement of science

This research has some limitations. These include the possibility of losing some cases due to the possible increase in late notifications of violence against people with intellectual disabilities after the collection period, given that the Notifiable Diseases Information System (SINAN) allows data to be included at any time; difficulties in inferring causality given that this is a cross-sectional study; the possibility of inconsistencies as this is secondary data from compulsory notification forms for violence; the regional nature of the facts, as there may be differences in the profiles identified in other regions of the country, given the large geographical size of Brazil and its multiculturalism, even though São Paulo is a metropolis with a very diverse population, with people from all regions of the country. However, these limitations do not compromise the findings because consolidated epidemiological data on the phenomenon is still scarce, especially in developing countries.

The study contributes to the advancement of science as it sheds light on a problem of great biopsychosocial magnitude in people with intellectual disabilities, who are highly vulnerable to violent practices. Recognizing the main characteristics of victims and perpetrators allows society and decision-makers to plan and implement violence prevention actions and policies, as well as assertive coping strategies for victims. It also expresses the need to reflect on intersectoral measures (education, health, public safety and others) to guarantee the human rights of people with intellectual disabilities and to expand the culture of peace and justice.

## Conclusion

The main characteristics of people with intellectual disabilities who were victims of interpersonal violence included being between childhood and adolescence, female, brown or black skin color, with low levels of schooling and heterosexual orientation. Because of their age, most of the victims were single. The aggressor was related to the victim in some way, with mothers and fathers committing cases of neglect and/or abandonment and psychological and/or moral violence. Cases of torture were committed by friends or acquaintances, as well as strangers. Sexual violence was also reported, with strangers and family members being the main perpetrators.

Men were the main aggressors, with the exception of neglect and/or abandonment, which was perpetrated by women, and they were aged between 25 and 29. The aggressions occurred at home and more than once, with varying motivations. In cases of sexual violence, rape was the most frequent and the health care received was focused on collecting tests and prophylaxis for STIs, with few.

referrals to security or victim protection agencies, guardianship councils and services aimed at guaranteeing human rights.

The number of reports of violence against people with intellectual disabilities in the period was high, but may not represent all of the incidents, given that many of these people find it difficult to report what happened and sometimes need help from others to seek help. The data reveals a latent problem that requires planning intersectoral actions to ensure the well-being and dignity of the victims, as well as reducing the occurrence of the problem.

Compulsory notification of violence is an important support tool for monitoring cases and helping politicians and public managers make assertive decisions for the target public. It is suggested that health professionals who care for victims of violence be trained on how to fill in the notification form correctly, as well as on specific lines of care for each case, such as for people with intellectual disabilities. There is a need for longitudinal or experimental research to better understand the temporal relationships between the variables of interest in the study of interpersonal violence, as well as the possible replication of the study in other scenarios (states or municipalities).

## Data Availability

The data sets obtained and/or analyzed during this study are publicly available. Information on the protocol, variables and data collected can be accessed at https://osf.io/4h68x/. Additional information can also be requested from the authors.

## Abbreviations

STI: Sexually Transmitted Infections
STROBE: Strengthening the Reporting of Observational Studies in Epidemiology
SINAN: Notifiable Diseases Information System
AMA: Ambulatory Medical Care
SNVS: National Health Surveillance System
DATASUS: Department of Informatics of the Unified Health System
SMS: Municipal Health Department
SP: São Paulo
IGN: Ignored
EF: Elementary School
HIV: Human Immunodeficiency Virus
IBGE: Brazilian Institute of Geography and Statistics
